# The impact of public leadership on collaborative administration and public health delivery

**DOI:** 10.1186/s12913-023-10537-0

**Published:** 2024-01-23

**Authors:** Muhammad Zia ud din, Xu Yuan yuan, Naqib Ullah Khan, Christophe Estay

**Affiliations:** 1 School of Public Administration, Central South University, Yuelu District, Changsha, Hunan 410017 China; 2 FERRANDI Paris (France), Lirsa, Cnam, Hesam Université, Paris, France

**Keywords:** Public leadership, Collaborative administration, Public health delivery, Social information processing theory, Collaboration theory

## Abstract

**Background:**

This research depicts the linkage of public leadership on public health delivery (PHD) and collaborative administration. The research is also focused to examine the effect of public leadership on public health delivery through the intervening variable of collaborative administration by using both social information processing theory and collaboration theory.

**Methods:**

This research is based on quantitative method. Data was collected from 464 public hospital administration in the context of Pakistan. This study evaluated data using SPSS, AMOS, and PROCESS Macro.

**Results:**

Public leadership has a positive profound effect on public health delivery and collaborative administration, and that collaborative administration significantly promotes public health delivery. The outcomes also exposed that public leadership has substantial influence on public health delivery through intervening collaborative administration.

**Conclusions:**

Whilst public leadership demonstrated positive outcomes on public health delivery and collaborative administration, there is a need for more rigor studies on collaborative governance leadership, collaborative ethics and collaborative norms in the public health service.

**Supplementary Information:**

The online version contains supplementary material available at 10.1186/s12913-023-10537-0.

## Introduction

The transition in the global economy and the dynamic environment have presented public health organizations with a challenge to enhance public health delivery [1]. The WHO report concluded that boosting effectiveness of public services is the first step toward raising public health institutions performance [2]. Despite the widespread agreement on the significance of government health provision, little is known about how leadership operates in the public health sector [3]. The “publicness” of health organizations creates a unique setting that is unlike any other type of organizations (Private, corporations and NGO’S) for their leaders [4]. Public health organizations often come under fire for being overly bureaucratic, static, unmoving, and conservative as compare to private health hospitals [5]. Public leadership practices may change public health organizations with their public health delivery in developing countries [6]. As strong hierarchical leadership seen collaborative administration is either undesirable or ineffective in bringing parties together in collaborative process [7]. It is believed that collaborative administration will bring together administrative stakeholders and engage them through Public leadership [8].

“Public leadership is a type of leadership specific to the public sector organization with new aspects including accountability, rule following, political loyalty and network”. So, PL is a powerful and effective kind of leadership uniquely suited to the government institutions. Public Leadership interventionist mediation techniques are essential for collaboration among all stakeholders [9]. It is stated that public leaders often intervene in a more direct way to pursue collaboration agenda [10]. Therefore public leadership is essential for embracing, empowering, involving, and mobilizing public health sector in order to progress towards collaboration [11]. Moreover, it is argued that public leaders “give meaningful voice to participants” and encourage them to attend to one another. Public Leaders ensure creativity by synthesizing the diverse knowledge of hospital administration to generate new concepts and comprehension [12]. Researchers in this field have long argued that further empirical studies are needed to determine the connections between public leadership and other factors influencing public health organizations, such as employee motivation and turnover [13–15].

While collaboration literature emphasis on governance which stated that collaboration is delivered by the public institution officials [16]. Collaborative administration is one of dimension of the collaboration, in which “partner organizations rely on administrators/managers to collaborate, mitigate conflicts, establish formal communication channels, agree on mutual goals in the collaboration activities” [17]. So, it is supposed that the public organization specifically hospital administration also ensures intra and inter organizational collaboration through observing the above-mentioned process of collaborative administration. As public hospitals administrators/managers are challenging task and expose to the collaborations internally and externally. So, the focus on the public hospitals’ administrators/managers collaborations with other public hospitals administrators/managers to ensure effective PHD.

According to the Social Information Processing (SIP) theory, people’s beliefs and actions are manipulated by the information they process from their social environments [18]. This theory suggested that because leaders have the most influence and authority in an organization, those who report to them actively obtain information from their leaders’ actions. Those subordinates process that information from their leader in their own way [19]. Using this theory, it is argued that the public leadership behaviours information is processed by hospitals administrators/managers for successful public health services delivery. With the help of SIP theory, public leaders may shape the mindsets and actions of public hospital administrators/managers through information sharing For example, public leadership enables the administration of public hospitals to effectively process information pertaining to the accountability aspect, communication and knowledge sharing, which in turn would allow subordinates to remove accountability barriers in public hospital administration for effective PHD [20]. Public health delivery goals may be more easily attained if public hospital administration is motivated to follow action plans by rule-following aspect of public leadership [21]. Public hospital administration may be motivated to work hard to achieve public health goals by the political loyalty of public leaders [22]. As part of public leadership dimension, network aspect allow administration to encourage managers and administrators to work in collaboration with partner organizations and external stakeholders to acquire the information and resources needed to carry out effective public health delivery [23]. We postulated that the assessment of public leadership qualities (including accountability for actions, adherence to regulations, democratic commitment, and participatory administration) by hospital administration could potentially enhance the provision of public health services following SIP principles. As a result of complex and dynamic nature of pernicious public health issues—which encompass challenges across sectors, multiple levels, and actors—they cannot be consolidated within the a single public hospital [24–26]. By involving other Stakeholders of the community, public hospitals can determine collaborative value, explain factors which influence that value, and explore which kinds of strategic resources are needed to affect community through collaborative administration [27]. In order to explain ‘how to’ put collaborative administration into practice and to create sustainable health results, the literature highlights the need of ‘informal variables’ (such as facilitative leadership, trust, commitment, common understanding, and values) [28]. Good intentions and following established procedures can only become true collaborative administration if they are put into practice.

Therefore, ‘public leadership’ and ‘collaboration’ are essential for public health institutions; however, a dearth of specificity and nuance in theory and empirical research hinders the development of knowledge and understanding regarding public leadership for collaborative administration.

Previous studies have demonstrated that leadership perceptions affect public health delivery in two ways:, by affecting collaborative administration procedures directly and by affecting public health delivery itself indirectly [29]. The collective impact of public leadership on public health delivery can be explained by the SIP theory [30]. This theory also effects public leadership directly and their subordinates indirectly. So, SIP Theory support this study as managers or administrators process information from their organization leader. collaboration theory illustrates that the managers and administrators collaborate with multiple stakeholders for effective public service delivery.

Considering the increased emphasis on open and honest sharing of actions and communications within collaborations and with other actors, the accountability aspect of PL may have an effect on the collaboration building processes (goal setting, role clarification, social relationships, and decision making). Employees’ ability to manage interpersonal conflicts and problems, as well as their individual and collective duties and obligations, may be influenced by the rule-following part of public leadership. Because it strengthens them to resolve to stick together for the greater good. The political loyalty aspect of PL may have an impact on the interpersonal relationship and problem-solving aspects of collaboration building. The network facet of public leadership can have an impact on the collaborative building processes (e.g., goal formulation, social relations, and issue solving) by enabling and motivating various stakeholders to operate in network and partnerships internally and externally. According to this, the establishment of hospital administration collaborations can be affected by all characteristics of PL, which is illustrated as PL effect on project success through teambuilding in Pakistani context [31].

This study utilizes both SIP theory and collaboration theory to inquire into the relationship between public leadership, collaborative administration and health delivery. Moreover, the study also examines how collaborative administration acts as a mediator between public leadership and public health delivery. This research adds to the existing literature on public leadership by considering public health delivery and collaborative administration in the context of strengthening government health institutions. So, following are research questions.

- What is relationship between public leadership with public health delivery?
- What is relationship between public leadership with the collaborative administration?
- How collaborative administration mediates between the relationship of public leadership and public health delivery?

## Theoretical background and hypotheses development

### Public leadership and public health delivery

International governments plan and implement public health services for numerous socioeconomic purposes [32]. The GOP allocated annually budgets for thousands of public health programmes [33]. The political leadership usually comes up with the idea for these public health delivery programs, which are then formulated by the central government and carried out by the local government. Political leadership eventually entrusted public leadership and their public hospital administration for implementation for public health delivery. Success in effective public health delivery requires the participation of many groups and individuals, including public health institutions, political leadership, health planning institutions, public health officers, private hospitals, trust hospitals, community health organizations, social security hospitals and the general public at large [34]. Several scholars agree that a public health delivery is successful if it is carried out successfully, produces the desired results, helps the health organizations, creates room for forthcoming development and believes for effective public health delivery to all actors [35]. Failure and success in public health delivery is contingent on a number of factors [36]. However, leadership behaviour is recognized as a significant factor that determines success or failure [37]. According to a study poor public leadership style is responsible for 80% of public health failures, The extant empirical evidence suggests that different traditional leadership styles have varying impacts on public health delivery [38, 39]. For instance, transformational-leadership influences successful public health delivery by empowering, motivating and fostering collaborations [40]. Ethical leadership by fostering an environment in which employees adhere to high health ethics and standards [41]. A servant leader assists, cares for, and authorizes health specialists [42]. Inclusive leadership through the creation of an inclusive setting and prospects in which all stakeholders participate and receive the leader’s support [43]. Conventional leadership behaviors and efficient public health delivery are supported by social behavior theories like SIP and collaboration theory [44–46].

According to researchers of public health management, there is a need of different set of leadership behaviors than those are in public sector for effective public hospitals administration performance [47]. Using prior research and the relationship-based perspective on leadership. It is conceived as four-dimensional construct based qualities of competent public hospital administration as leaders [48]. Whereas (1) accountability leadership dimension of PL extended open and unequivocal communication among organizational stakeholders. (2) Norms and processes are emphasized heavily in rule-following leadership aspect of public leadership. Thirdly, political loyalty is focused on supporting political leaders and keeping in good standing with them (4) Network leadership aspect of public leadership encourage administrators to network both inside and outside the government [49]. The three facets of PL describe the central features of public hospitals in the government; the fourth, NL, was introduced to reflect the increasing significance of working in collaborating within government organizations [50]. Previous research has shown that public leadership is linked with an increase in personnel institutional commitment, engagement, motivation, citizenship behavior, and change orientations [14], professionalism in teaching profession and effectiveness in public health organizations [51]. As postulated by the social information processing theory and collaboration theory, public leadership has beneficial impact on employee motivation and productivity [52]. The limited empirical literature on public leadership highlights the need for more empirical studies examining the impact of public leadership on a wide range of public context variables [15]. The effect of public leadership on public health delivery is an area where the existing literature is lacking in particular. In SIP theory, public leadership is assumed to influence public health delivery in a variety of ways; for example, the accountability facet of PL may improve sharing specialization and actions with multiple stakeholders, thereby aiding them in resolving public health issues and implementing public health delivery actions professionally. Sharing of knowledge could facilitate the correction of procedures for the efficient implementation of public health plans by administrators [53] as well as increase stakeholders participation [54]. Researches indicate that the perception of enhanced performance in public hospitals is linked to the perception of increased accountability in public health institutions [55]. Public health delivery objectives can be achieved through the rule-following part of PL, which can persuade stakeholders to perform in accordance with prescribed public health actions and processes. Following these strategies and procedures could result in the successful completion of public health delivery programmes [56]. Rule abidance can more effectively align stakeholders’ actions with public health objectives, persuading tasks efficiently and in accordance with established standards [57]. Multiple stakeholders may be influenced by the political loyalty facet of PL to be faithful and committed in defending policies of political leaders, which can only be defended when the public health delivery is effective [11]. Public health Mangers/administrators loyalty has been identified as a key component in boosting public health delivery in public hospitals [58].

#### H1:

PL is positively associated with public health delivery.

### PL and collaborative administration

Effective public sector executives, according to some scholars, are less materialistic, open and mindful and more participative rather than directive [59–61]. These public sector executives played pivotal role in policy implementation through public leaders behaviors. It is argued that public leaders play a critical role in encouraging multi stakeholders to collaborate to solve complicated public problems including public health problems [62]. So, PL is conceptualized concept for public organizations that prioritizes the public goods delivery and generates value of public organizations in the public [13].

Historically, public health administration has devoted insufficient attention to the topic of leadership [63]. This is especially true for public health sector public leadership [64]. Most modern treatments of public leadership concentrate on intraorganizational leadership [65–67]. In other words, the modern emphasis in public health administration is on cultivating leaders evolves within the context of organizations “often operate across organizations as well as within hierarchies” [11, 68]. Various personal attributes, skills and behavioral competencies are required for public leadership in collaborative context of public health management [69]. Previous studies on collaboration, especially collaboration for public services, demonstrates critical role of leadership in determining the success or failure of collaborative projects [70, 71]. It is believed that “public leadership makes a huge difference” and influenced by his collaborations traits with other stakeholders in hospital administration [72]. Public leadership in public hospitals adopts collaboration through unified efforts across diverse and often conflicting groups in the pursuit of a health delivery objective [73, 74]. Traditional notions of leadership are organizational (hierarchical) and ultimately centered on authority and motivating “followers.” However, public leadership for collaboration is radically different. Thus, the argument here is that effective leadership across public organizations requires additional competencies. Several scholars have developed public management-applicable models of collaborative (sometimes termed “facilitative”) leadership [75–78]. The majority of studies examine leadership from the perspective of governance [79] but collaborative administration is one of the dimension of collaboration carried out by public officials [17]. As public institutions governance are carried out by administrators/Mangers [80] Since the concentration is on public leadership as a process of bringing together stakeholders to solve public health problems, none of the examples are explicitly from the public health sector and collaborative administration. In other words, rather than viewing public leadership in terms of achieving PHD, researches emphasized on addressing human resources and psychological practices of health professional like knowledge sharing and public sector motivation in Pakistan [81, 82]. Therefore, leadership is exercised across all sectors, but public leadership is limited to only government organizations. Numerous traits, skills and actions have been singled out as essential for public leadership [83]. Public Leadership development in the public hospitals is broken down into 37 distinct competencies, including 10 personality traits, 6 “meta-skills,” and 21 actions. These are detailed in the book “Dynamics of Leadership in the Public sector” [84]. The network characteristic of PL may stimulate and allow health managers/administrators to cultivate new relationships internally and externally, thereby enhancing coordination among stakeholders [85]. This facet of public leadership may inspire networking among stakeholders, which may persuade in apportioning necessary capital and skills to accomplish tasks [86]. Suggesting SIP, we hypothesize that the combination of the characteristics of PL (AL, RL, LL, and NL) can influenced attitudes of public hospital administration towards achieving public health delivery outcomes. The objective here is to align the public leadership skills applicable to the collaborative administrative context. Above discussion draws following hypothesis.

#### H2:

PL is positively associated with collaborative administration.

### Collaborative administration and public health delivery

The ‘wicked’ challenges in public health delivery have prompted scholars of public health administration to call for a greater emphasis on establishing collaborative administration systems [87–89]. By concept, public health services are inter-organizational activities that require all actors, including patients, to collaborate to create public health value [90]. “Collaborative administration” can help public-sector institutions articulate public value, its drivers and the strategic resources needed to improve community health outcomes [91]. By facilitating the creation of ‘strong’ policies, this method of learning alludes to an outcome-focused viewpoint. Achieving community resilience and sustainable socioeconomic development by having all relevant stakeholders participate in the policy codesign, coproduction, and assessment processes in health sector [92, 93]. This perspective envisions a plural state where numerous interdependent players supply public health services and diverse procedures impact decision [94].

#### H3:

collaborative administration is positively associated with public health delivery.

### The mediating nature of collaborative administration

**Fig. 1:**
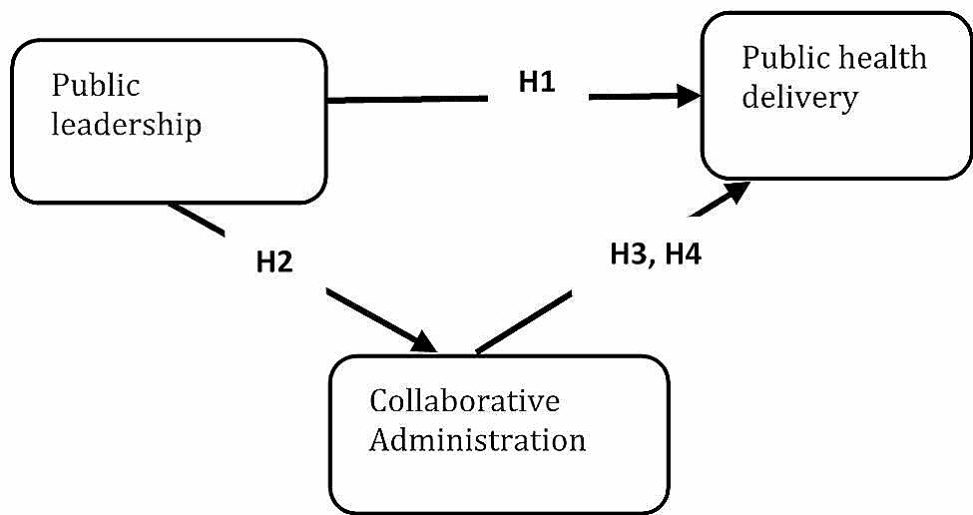
Conceptual model

“Collaboration administration is a strategy to help public officers to deal with public services issues by engaging with other governmental and non-governmental actors inside and outside focal organization to implement achieve policy objectives [95]. Particularly, collaboration can be one type of organizational arrangement for tackling wicked policy problems which require collective efforts from multiple public hospitals for delivering public health services which single public hospital may not be able to provide in a more efficient way [96]. Such intergovernmental arrangement can enhance public health administration capacities and desired policy implementation. In public health literature, collaborations outward including other governments and non-governmental stakeholders could make substantive impacts on public health policy outcomes pertaining to public health delivery [97]. It is indicated a positive association between managerial leadership and public health Collaboration in survey [98]. Other scholars also observe a similar pattern in the realm of health services and community services [99]. This can occur because collaboration administration could stimulate policy knowledge, resources, supplementary skills, community support and trust in public hospitals administrators/managers across public hospitals. This will contribute to the accomplishment of public hospital administrators/managers responsiveness that can only be obtained by collaborating with other public hospitals, rather than sole health services provided by single public hospital [100, 101]. It is worth noting that collaborative administrative patterns could lead to specific public hospital networks [102]. For public hospitals, building strong and weak collaborative administration can serve for different public hospitals facilities and equipment’s to accommodate patients [103, 104]. A particular collaborations pattern (collaborative administration) could push performance upward or downward due to the type of resources transmitted via collaborations which may or may not be suitable to mitigate uncertainty and boost health services reputation [105]. These findings suggested that public hospitals would need to strategically select other public hospitals which would in turn facilitates the patient’s public health issues. On other hand, urban studies recognized the importance of intergovernmental collaboration in health delivery [106]. The main rationale behind this type of administrative collaboration can be rectification of health care organization scope and services mismatch [107], deterioration of fiscal conditions [108], improvement of public health quality or sometime a bunch of these together [109]. If collaborative administration can improve public hospital performance, we then expect public leaders in public hospitals would consider it in health arrangement as a viable option. Indeed, Public hospitals are one of major type which provide public services that oftentimes encountered issue of diseconomies of scale in emergency situation [110]. Despite the collaboration barriers among public hospital administration/Managers, local officials see public health delivery economy of scale [111]. Given the influences of administrative collaboration on public health delivery, the question of interest would then turn to be what is relationship between public leadership, collaborative administration and public health delivery might be. As illustrated that public leadership can modify the preference of public hospitals on the choices of collaborative administration which would then change public health delivery. Due to different education and managerial background as well as career goals, hospitals administrators/managers may assign different values to administrative decision making. For example, unban public hospital administrators/managers with progressive ambitious are more likely to be the seller and less likely to be buyer of public health delivery [112]. Self-development minded public hospital administrators/managers are more inclined towards collaborative administration [113]. As a result, public leadership can open the opportunity window for building collaboration and bring changes in traditional administrative practices in the public hospitals context. Viewing public health services issues in intra and interorganizational perspective, public hospitals might seek for collaboration as alternative to enhance the level of health delivery [114]. Given this empirical evidence, it is supposed that collaborative administration can be directed through public leadership which can in turn affect public health delivery. so, it is hypothesized that: (See Fig. 1)

#### H4:

collaboration administration intervenes the linkage between PL and PHD.

## Materials and methods

### Sample and procedure

The data utilized in this study was gathered via an online descriptive survey. By applying this scientific method to this survey research, source materials were rigorously analyzed and evaluated, data were interpreted, generalized and predictions were made. Since the purpose of this research was to examine the public leadership and administrative collaboration of administrators /managers in public hospitals. As a result, the descriptive survey method of research was suitable for this research as emphasized by [115]. We initially approached health department of Punjab Province to apprise them of the research’s objectives. The policy formulation and oversight of hospital administration/managers at 88 THQ and 34 DHQ public hospitals throughout the province fall under the purview of health department Punjab. With the consent of the Health Secretary of Punjab, we have obtained permission to carry out the survey. To assist us in obtaining the personal information and email addresses of medical unit heads and administrators/managers DHQ and THQ of public hospitals, he rendered the services of his subordinate. Thus, the data were gathered from the administrators/managers of 34 DHQ, 88 THQ, public hospitals and the heads of their respective medical units. The survey link response and request letter have been dispatched to the email addresses of the administrators/managers and their respective medical unit heads of public hospitals. In the request-response letter, emphasis was placed on the anonymity of the responses and study’s objectives.

The online Google form survey was designed in such a way that we have put first conditional option as: have you collaborated with other hospital administration recently (in the past two years)? Clicking “Yes” allowed the respondent to the next section. We have put two options in the next section option 1 was “public leadership (topmost head)”, and option 2 was “subordinate to the public leadership (department head)”. Clicking the first option was leading the hospital head to respond to collaborative administration, public health delivery and public leadership general information, demographics, and his/her name. Clicking the “subordinate to public leadership (head)” option was leading subordinate departmental head in the hospital to measure the public leadership questionnaire, the name of the departmental head, departmental-related general information, and demographics of respondents. Following this process, we have received 496 valid responses from hospital head and 484 valid responses from subordinate hospital departmental head. Using the hospital names, 464 responses of the subordinate departmental head were matched with the responses of their hospital leader (head). The findings of the study are based on total valid response rate of 73%. The potential demographic control factors of 464 hospital wards head that may influence public health delivery are presented in the following (Table 1).

**Table 1:** Description of demographics variables

Description	Measures	F	Percent
Gender	Male	321	69.1
	Female	143	30.9
Age	20–30	95	20.5
	31–40	119	26.4
	41–50	163	34.6
	50–60	87	18.5
Medical education (years)	Below 16	76	16.4
	16	218	46.9
	18	87	18.8
	other	83	17.9
Experience level (years)	Below 5	41	8.8
	6–10	52	11.2
	11–15	82	17.7
	16–20	95	20.4
	21–25	113	24.4
	Above 25	81	17.5
Total	464		

In order to assess the constructs, this research used existing developed measures. Participants were given a five-point Likert scale to score their level of agreement, extending from “1” for strongly disagreeing to “5” for strongly agreeing. 11-items were used to rate public leaders by adapting already existing scale developed for public leadership with its four aspects (accountability, rule following, political loyalty and network) by (Vogel, 2020) [116]. Cronbach’s alpha value 0.89 indicated that the research instrument had a high level of internal consistency. The 22-item SERVQUAL instrument with dimensions ( tangibility, reliability, responsiveness, assurance and empathy) developed by (Zeithaml, 1988) was adapted in this study to measures public health delivery [117] and is already used in public health services in Pakistan [118]. The Cronbach’s alpha coefficient for the reliability of the instrument utilized to evaluate the public health delivery was determined to be 0.92, indicating a high level of internal consistency.

To measure collaborative administration, this study used existing developed 11-item developed by (Thomas et al., 2009) [17], With a Cronbach’s alpha coefficient value of 0.91. Reliability analysis showed strong internal consistency. We used the gender, age, education level, and experience of public hospital administrators /managers as a control. It is because these control factors have the potential to influence the dependent variable (PHD) [119, 120].

### Data analysis

This study evaluated data using SPSS, AMOS and PROCESS Macro. The goal of the CFA was to assess the congruence between the empirical data and the hypothesized latent constructs. Latent constructs are accurately modeled by the observable variables as depicted by CFA results [121] (Table 2).

**Table 2:** Descriptive statistics, correlation, reliability, and validity

Variable	CR	AVE	Mean	SD	PL	PHD	CA
PL	0.89	0.67	3.6458	0.8251	0.77		
PHD	0.92	0.64	3.8542	0.7154	0.29**	0.78	
CA	0.91	0.61	3.6527	0.7869	0.28**	0.38**	0.79

*Note(s)*: N = 464 with **p > 0.01., the diagonal depicts the AVE, whereas the off diagonal represents correlations. PL, PHD, CA, CR, AVE, SD representing public leadership, public health delivery, collaborative administration, composite reliability, average variance extracted and standard deviation respectively

we then tested our hypothesis by using linear regression analysis with the mediation model-4. Using a linear regression model, the relationship between the explanatory and response variables was investigated. The mediation model-4 test was utilized to evaluate the hypothesized mediator’s effect relationship between explanatory and outcome variables. The aforementioned tests facilitated the determination of the significance and strength of the hypothesized associations, as well as the influence of the mediator variable on the relationship between the independent and dependent variables [122] (Table 3).

**Table 3:** Model fit indices

M	Spec.	χ2	df	χ2 /df	CFI	TLI	SRMR	RMSEA
M1	CFA outcome	669.58*	28	25.542	0.69	0.56	0.08	0.26
M2	CFA outcome	815.16***	515	1.643	0.94	0.92	0.04	0.05

*Note(s)*: *P < 0.05 and ***P < 0.001

## Results

### Construct factor analysis, reliabilities and validities

Using confirmatory factor analysis (CFA), the suitability of the three-factor model consisting PL, public health delivery and collaborative administration was evaluated. The analysis showed that the three-factor model demonstrated a good fit with the data (χ2 = 815.16, df = 515, χ2 /df = 1.643, CFI = 0.94, TLI = 0.92, SRMR = 0.04, RMSEA = 0.05) than the single factor model, which showed poor fit (χ2 = 669.58, df = 28, χ2 /df = 25.542, CFI = 0.69, TLI = 0.56, SRMR = 0.08, RMSEA = 0.26) (Table 2). The greatest emergent component explained 32% of variation, below Harman’s single-factor statistical threshold of 40% [123]. This suggests that common method bias (CMB) has a relatively small effect on self-reported data. Cronbach’s alpha scores displayed in above-mentioned section (Measures) demonstrated that study’s constructs are very reliable. Furthermore, all structures had CR that were greater than 0.80 (Table 4), demonstrating study’s constructs high degree of internal consistency among them [124]. AVE was employed to assess convergent validity of constructs. AVE values over 0.50 showed that the constructs in the study had good convergent validity. Therefore, the study constructs have strong convergent validity. Fornell and Larcker created method used to evaluate discriminant validities [125]. The results depict (AVE) were greater than correlations between constructs correlations, supporting discriminant validity. It also revealed that construct correlations are smaller than the square root of the AVE for each construct as depicted in (Table 4). This demonstrates the constructs’ discriminant validity [126].

**Table 4:** Regression results

Path			(β)	SE	t	
Controls						
Gender		PHD	0.06	0.08	−0.75	
Age		PHD	0.03	0.06	0.37	
Education		PHD	−0.01	0.07	0.15	
Experience		PHD	−0.16	0.09	−1.82	
Main effects						H
PL	→	PHD	0.28***	0.05	5.31	H1
PL	→	CA	0.27***	0.08	3.63	H2
CA	→	PHD	0.29***	0.06	4.68	H3
PL→CA	→	PHD	0.15***	0.05	3.09	H4

*Note(s)*: N = 464, N, x, PL→CA and ***p < 0.001 depict the number of respondents, interaction term, mediating impact of PL and PHD through CA and significance levels respectively. 5000 bootstraps and 95% confidence yielded these results. level

Given these figures and the orientations of study’s variables’ correlations, regression analysis seems reasonable to test these presupposed linkages. Regression analysis seems acceptable given these results and the study’s variable correlation as explained (Table 4).

### Regression results

Hypothesis are tested through SPSS PROCESSMACRO’s for linear regression and mediation model-4 [120]. Hypothesized associations are depicted in (Table 2). When controls were exhibited in the regression analysis (Table 3), PL exhibited a positive association with PHD (β = 0.28, p < 0.001) and CA (β = 0.27, p < 0.001). H1 and H2 demonstrated regression outcomes. The intervening variable collaborative administration was significantly associated with public health delivery (β = 0.29, p < 0.001), supporting H3 (Table 3). Thus, the research model’s paths a, b, and c are significantly connected. When all three routes in a mediation model-4 are considerably linked, it showed that model have a mediation effect [127]. To assess the mediating role of collaborative administration between public leadership and public health delivery, mediation model-4 was used for mediation analysis with 5000 bootstraps and 95% CI. It also depicted an indirect effect of PL on the PHD via CA (β = 0.15, p < 0.001) (Table 3). Based on prescribed criteria, it is observed that the bootstrap intervals, specifically [0.25, 0.48], do not include the value of 0. Consequently, it can be inferred that the mediating effect is significant [120]. Public leadership’s impact on the PHD has weakened but remained significant showing partial mediation, providing support to H4. The indirect effect was also verified through Sobel’s test [128]. The values of the product of coefficient were (SE = 0.05, t = 3.09, p < 0.001), all these support H4. Thus, empirical studies validated all study hypotheses H1, H2, H3, and H4 (Table 3).

## Discussion

This study addressed the relationship among public leadership, collaborative administration and public health delivery. Studying how public leadership affects public health delivery through intervening collaborative administration was another goal. The results indicated that public leadership is correlated with public health delivery. That is, more positive evaluations of public health delivery are recorded after a greater emphasis was placed on public leadership qualities. Previous studies corroborate this conclusion by showing the association between PL and project success in Pakistan is positive [31]. Strategic leadership styles also influence public health delivery in private health care contexts [129]. So, it stands to reason that “PL is a government institutions spcific leadership style” that is also linked with PHD by public hospitals administration explicitly [14]. Previous quantitative research has demonstrated that this public leadership is positively associated with the teachers’ competence [130], school education effectiveness [131], public sector motivation [132] and health performance [133]. This scholarship also showed that PL has a favorable effect on collaborative administration. That is to say, how people view collaborative administration is affected by the traits of public leaders. This conclusion is backed by studies that analyzed how leadership styles affected collaboration in environment management [134]. Earlier empirical research on collaborative administration and managerial leadership is reported not only from Pakistan but also from other countries [135, 136]. In addition, prior empirical research has demonstrated that PL effects civil servants commitment, workplace engagement, performance, citizenship behaviour and change orientations [30]. It is inferred from data analysis that public leadership promotes on collaborative administration. In other words, people’s impression of public health delivery is influenced by the public hospitals administrators/ managers collaboration. This finding from Pakistan’s collaboration context generalizes previous findings on interagency collaboration in health sector projects in Pakistan [137]. In addition, this study illustrated collaborative administration as a mediator between PL and PHD. Earlier research also demonstrated that PL effects the institutional administrative performance [138] academics [139], institutional change orientation [140]. From this research, it is deduced that collaborative administration plays a pivotal role in improving public health delivery through health administrators/managers in Pakistan.

### Novel contributions

This study adds to the body of literature by adding theoretical knowledge. First, the existing body of empirical research pertaining to the connection between public health delivery and public leadership behaviors is notably limited. Second, in terms of its influence on employees and organizational outcomes, the concept of PL is a relatively new phenomenon that has not been the subject of extensive empirical research [141]. Consequently, this research broadens the scope of PL by identifying collaborative administration within the framework of PHD. Third, collaborative administration serves as the intermediary connecting PL and PhD. Forth The application of the distinctiveness generof unique collaboration dimension (collaborative administration) in the context of hospital administration in Pakistan.

This study makes a significant theoretical contribution to the extant body of literature. Firstly, the existing body of empirical research on the correlations between managerial leadership behaviors and public health delivery is notably limited [142, 143]. Secondly, the concept of public leadership is a recently developed idea that has not been well studied in terms of its effects on employees and organizational outcomes [14, 15]. The primary notable contribution of this work is the establishing of a causal relationship between PL and PHD. Therefore, this study expanded the application of public leadership behaviors to the administration of public hospitals. The field of public management lacks sufficient literature on collaborative administration research, as emphasized by [17]. Specifically, there is a dearth of empirical studies examining the relationship between public sector-specific leadership approaches, such as public leadership and collaborative administration building within public health institutions. The third theoretical contribution of the research is an examination of empirical evidence linking collaborative administration and PL. This discovery further applies the notion of public leadership to collaborative investigations within the field of public management. Nevertheless, organizational outcomes and empirical findings regarding collaborative administration are not entirely consistent, according to a recent systematic review [144]. The identification of a substantial connotation between PHD and collaborative administration in this study strengthens the validity of the prior empirical findings. This result provides additional support for the notion that collaborative administration is a crucial CSF for PHD. Additionally, this study contributes to the current body of knowledge by illustrating that public leadership influences public health delivery not only directly but also indirectly through collaborative administration. Therefore, the findings of our research hold significance for scholars who are interested in determining the consequences of public leadership, the determinants of effective PHD and collaborative investigations in public management.

### Theoretical and policy implications

The practical implications of this study are substantial within the realm of public health delivery. The Public policy for public health is authorized and funded by legislative approvals, while the provincial health department is responsible for their planning and implementation. These are further supervised by administrators/managers of public hospitals and the heads of respective hospitals medical units’ heads in the public’s socioeconomic interest. All stakeholders aim for the success of PHD, prioritizing efficient response, desired outcomes, health advantages and the satisfaction of key stakeholders [145]. Based on this empirical finding and, it is strongly recommended that key stakeholders, such as administrators and managers of public hospitals give priority to the creation of efficient collaborative administrative procedures to ensure successful PHD. Public hospitals administrators/managers necessitate active participation and effective collaboration among subordinates to establish and attain shared health delivery goals. It requires promoting shared responsibilities, establishing clear individual roles, and adhering to organizational standards. Furthermore, fostering support in the development of robust interpersonal connections and improving problem-solving capabilities are integral elements of effective collaborative administration endeavors [146]. Administrators and managers of public hospitals and human resource (HR) divisions have the ability to improve collaborative administration through the implementation of an extensive array of training and development programs. As this research results indicated attributes of public leadership influence PHD and collaborative administration. we encourage public health planning departments to ensure leadership attributes of accountability, rule-following, political loyalty network governance in public hospitals administrators/managers, as these public leadership attributes manifest in the form of desirable employees and organizational outcomes. Furthermore, public hospitals administrators/managers are highly encouraged to practice accountability, rule following, political loyalty, and network governance leadership attributes more often than usual as these lead to collaborative administration and PHD. Human resources departments are advised to prioritize the recruitment of administrators/ managers at public hospitals who possess strong inclinations toward accountability, adherence to rules, political loyalty, and network-oriented attributes. There is a greater likelihood that public employees who possess these characteristics will contribute significantly to the efficient operation of PHD. In addition, such employees assist in collaborative administration for effective and productive PHD.

### Limitations of the study and future research directions

We collected data from the study using the commonly accepted online self-reporting descriptive survey method. This method has both strengths and weaknesses. One notable advantage of this methodology is that it efficiently enabled us to gather data from 464 administrators/managers of public hospitals and their respective medical units’ heads. It allowed us to measure public leaders’ opinions of PHD and collaborative administration, and medical wards head perceptions of the public leadership attributes through responding to self-report measures. Social desirability bias is the most significant drawback of self-reported instruments, as respondents are likely to provide biased responses regarding their PL, collaborative administration practices and PHD. Nevertheless, this potential bias was reduced by consistently emphasizing that the study’s objective is academic in nature and that all responses to the measures are maintained in strict confidentiality. Secondly, self-reporting measures sometimes cause common method bias (CMB) [147]. Risk of CMB was mitigated through the accumulation of data from two sources: medical units of hospitals assessed the public leadership attributes of public hospital administrators/managers, while the administrators/managers responded to measures of collaborative administration and PHD. Furthermore, single-factor statistics developed by Harman indicate that the data lacks CMB. It is recommended that future investigations employ consistent self-reporting instruments at various time intervals in order to bolster the reliability and consistency of the associations between the variables. The findings presented herein are derived at the variable level of the model. Nevertheless, it is possible that the dimensions of public leadership—namely AL, RL, LL, and NL—have an alternative impact on the dimensions of collaborations (such as governance, mutuality, autonomy, and norms) and PHD (including tangibility, reliability, responsiveness, assurance, and empathy). Subsequent investigations may explore the precise attributes of the variables in order to attain more profound understandings of the phenomenon. Additionally, a mixed-methods approach may be utilized to bolster the validity of the research model’s conclusions and fortify the validity of the results.

## Conclusions

The objective of this research endeavor was to examine the impact of collaborative administration on PHD and to assess the influence of public leadership on PHD. The investigation also sought to examine the impact of collaborative administration as a mediating mechanism between public leadership and PHD. Data was collected from public hospitals administrators/ managers and their immediate medical units’ heads in the public health management context of Pakistan. Results revealed that public leadership improves PHD and collaborative administration, and that collaborative administration improves PHD. Results also revealed that public leadership engenders collaborative administration which ultimately improves PHD.

### Electronic supplementary material

Below is the link to the electronic supplementary material.

**Supplementary Material 1**: Questionnaire

## Data Availability

The datasets generated during and/or analyzed during the current study are available from the corresponding author on reasonable request.

## Abbreviations

PL: Public Leadership
CA: Collaborative administration
PHD: Public health delivery
