# Supplementary material for: The impact of public leadership on collaborative administration and public health delivery
Source: BMC Health Serv Res. 2024 Jan 23;24:129. doi: 10.1186/s12913-023-10537-0 (PMC10807078; doi:10.1186/s12913-023-10537-0)
Supplement: Supplementary file 1 — Supplementary Material 1: Questionnaire [file 12913_2023_10537_MOESM1_ESM.docx]

| **Questionnaire** |
| --- |

**NAME CELL NO NAME OF Hospital:**

**Demographic section**

**What is your age bracket?**

20-25years 26-30 years 31-35 years 36-40 years 41-45 years 46-50 years 51-55 years 56-60 years

**What is your level of Education?**

Intermediate Bachelor Master MS/M. Phil other

**Hospital (ward)?**

physician cardiology surgery oncology other

**Section I I:** Please indicate the extent of your agreement with the following statement on a 5-point scale. (Please circle yyour answer)

| **1** | **2** | **3** | **4** | **5** |
| --- | --- | --- | --- | --- |
| ▼ | ▼ | ▼ | ▼ | ▼ |
| **Strongly**  **Disagree** | Disagree | Neutral | Agree | Strongly Agree |

| PL-1 | Do you agree that your hospital encourages you and you colleagues to explain your actions to various stakeholders.? | **1** | **2** | **3** | **4** | **5** |
| --- | --- | --- | --- | --- | --- | --- |
| PL-2 | Do you agree that your hospital encourages you to inform stakeholders of your way of working? | **1** | **2** | **3** | **4** | **5** |
| PL-3 | Do you agree that your hospital strives to ensure that you openly and honestly share the actions of your hospital unit with others.? | **1** | **2** | **3** | **4** | **5** |
| PL-4 | Do you agree that partner hospitals emphasize to you and your colleagues that it is important to follow the law? | **1** | **2** | **3** | **4** | **5** |
| PL-5 | Do you agree that partner hospitals give you and your colleagues the means to properly follow governmental rules and regulations? | **1** | **2** | **3** | **4** | **5** |
| PL-6 | Do you agree that all partner hospitals ensures that you accurately follow the rules and procedures? | **1** | **2** | **3** | **4** | **5** |
| PL-7 | Do you agree that your hospital encourages you and your colleagues not to jeopardize the relationship with political heads, even if that entails risks.? | **1** | **2** | **3** | **4** | **5** |
| PL-8 | Do you agree that your hospital encourages you and your colleagues to defend political choices, even if you see shortcomings.? | **1** | **2** | **3** | **4** | **5** |
| PL-9 | Do you agree that your hospital encourages you and your colleagues to invest substantial energy in the development of new contacts.? | **1** | **2** | **3** | **4** | **5** |
| PL-10 | Do you agree that Partner hospitals motivates you and you colleagues to regularly work together with people from our networks.? | **1** | **2** | **3** | **4** | **5** |
| PL-11 | Do you agree that your hospital motivates you and your colleagues to develop many contacts with people outside your own department? | **1** | **2** | **3** | **4** | **5** |
| CA-1 | Do you agree that your Partner hospitals (including your hospital) rely on a manager to coordinate the collaboration’s activities? | **1** | **2** | **3** | **4** | **5** |
| CA-2 | Do you agree that your hospital brings conflicts with partner hospitals out in the open to work them out among the hospitals involved?? | **1** | **2** | **3** | **4** | **5** |
| CA-3 | Do you agree that your hospital relies on an external authority to resolve conflicts with partner hospitals? | **1** | **2** | **3** | **4** | **5** |
| CA-4 | Do you agree that your hospital relies on formal communication channels when contacting partner hospitals about issues related to the collaboration? | **1** | **2** | **3** | **4** | **5** |
| CA-5 | Do you agree that your hospital has problems getting in touch with partner hospitals when you need to contact them? | **1** | **2** | **3** | **4** | **5** |
| CA-6 | Do you agree that You, as a representative of your hospital in the collaboration, understand your hospital’s roles and responsibilities as a member of the collaboration? | **1** | **2** | **3** | **4** | **5** |
| CA-7 | Do you agree that your Partner hospital meetings accomplish what is necessary for the collaboration to function well? | **1** | **2** | **3** | **4** | **5** |
| CA-8 | Do you agree that your Partner hospitals (including your hospital) agree about the goals of the collaboration?? | **1** | **2** | **3** | **4** | **5** |
| CA-9 | Do you agree that your hospital’s tasks in the collaboration are well coordinated with those of partner hospitals? | **1** | **2** | **3** | **4** | **5** |
| CA-10 | Do you agree that you feel partner hospitals keep an eye on your hospital’s activities to make sure you are doing what you are supposed to be doing in the collaboration?? | **1** | **2** | **3** | **4** | **5** |
| CA-11 | Do you agree that your hospital keeps an eye on partner hospitals’ activities in the collaboration to make sure they are doing what they are supposed to be doing in the collaboration?? | **1** | **2** | **3** | **4** | **5** |
| PSD-1 | Do you agree that your hospital have up-to-date equipment? | **1** | **2** | **3** | **4** | **5** |
| PSD-2 | Do you agree that your hospital physical facilities are visually appealing? | **1** | **2** | **3** | **4** | **5** |
| PSD-3 | Do you agree that employees of your hospital are well dressed and neat in appearance? | **1** | **2** | **3** | **4** | **5** |
| PSD-4 | Do you agree that your hospital have visually appealing materials associated with the service provision? | **1** | **2** | **3** | **4** | **5** |
| PSD-5 | Do you agree that when this hospital promise to do something by a certain time, they should do so? | **1** | **2** | **3** | **4** | **5** |
| PSD-6 | \| Do you agree that when patients have problems, these hospitals should be sympathetic and reassuring? \| \| --- \| \|  \| | **1** | **2** | **3** | **4** | **5** |
| PSD-7 | \| Do you agree that these hospitals should be dependable? \| \| --- \| |  |  |  |  |  |
| PSD-8 | Do you agree that your hospital have performs the service right the first time? | **1** | **2** | **3** | **4** | **5** |
| PSD-9 | Do you agree that your hospital Provide services at the time promised? | **1** | **2** | **3** | **4** | **5** |
| PSD-10 | Do you agree that your hospital maintains error-free records? | **1** | **2** | **3** | **4** | **5** |
| PSD-11 | Do you agree that your hospital informs public when services will be performed? | **1** | **2** | **3** | **4** | **5** |
| PSD-12 | Do you agree that your hospital offers prompt services to public? | **1** | **2** | **3** | **4** | **5** |
| PSD-13 | Do you agree that your hospital always willing to help public? | **1** | **2** | **3** | **4** | **5** |
| PSD-14 | Do you agree that your hospital readily respond to public request? | **1** | **2** | **3** | **4** | **5** |
| PSD-15 | Do you agree that your hospital able to instill confidence in public? | **1** | **2** | **3** | **4** | **5** |
| PSD-16 | Do you agree that public feel safe in their transactions with your hospital? | **1** | **2** | **3** | **4** | **5** |
| PSD-17 | Do you agree that employees of your hospital are courteous at all times? | **1** | **2** | **3** | **4** | **5** |
| PSD-18 | Do you agree that your employees have the knowledge to answer public questions? | **1** | **2** | **3** | **4** | **5** |
| PSD-19 | Do you agree that your hospital have convenient operating hours to all? | **1** | **2** | **3** | **4** | **5** |
| PSD-20 | Do you agree that employees of your hospital give personal attention to all? | **1** | **2** | **3** | **4** | **5** |
| PSD-21 | Do you agree that employees of your hospital have patients’ best interests at heart? | **1** | **2** | **3** | **4** | **5** |
| PSD-22 | Do you agree that employees of your hospital understand public needs? | **1** | **2** | **3** | **4** | **5** |
